# A Novel Method for Monocular Depth Estimation Using an Hourglass Neck Module

**DOI:** 10.3390/s24041312

**Published:** 2024-02-18

**Authors:** Seung-Jin Oh, Seung-Ho Lee

**Affiliations:** Department of Electronic Engineering, Hanbat National University, 125, Dongseo-daero, Yuseong-gu, Daejeon 34158, Republic of Korea; 30221175@o365.hanbat.ac.kr

**Keywords:** monocular depth estimation, hourglass neck module, swin transformer V2, masked image modeling, deformable attention

## Abstract

In this paper, we propose a novel method for monocular depth estimation using the hourglass neck module. The proposed method has the following originality. First, feature maps are extracted from Swin Transformer V2 using a masked image modeling (MIM) pretrained model. Since Swin Transformer V2 has a different patch size for each attention stage, it is easier to extract local and global features from images input by the vision transformer (ViT)-based encoder. Second, to maintain the polymorphism and local inductive bias of the feature map extracted from Swin Transformer V2, a feature map is input into the hourglass neck module. Third, deformable attention can be used at the waist of the hourglass neck module to reduce the computation cost and highlight the locality of the feature map. Finally, the feature map traverses the neck and proceeds through a decoder, comprised of a deconvolution layer and an upsampling layer, to generate a depth image. To evaluate the objective reliability of the proposed method in this paper, we used the NYU Depth V2 dataset to compare and evaluate the methods published in other papers. As a result of the experiment, the RMSE value of the novel method for monocular depth estimation using the hourglass neck module proposed in this paper was 0.274, which was lower than those published in other papers. The lower the RMSE value, the better the depth estimation method; therefore, its efficiency compared to other techniques has been proven.

## 1. Introduction

As the importance of the role of depth estimation technology increases in autonomous driving, AR (Augmented reality)/VR (Virtual Reality), drones, and robots, the need for research in the field of depth estimation is expanding. Prior to the activation of deep-learning-based depth estimation, various sensors are used to add depth values when taking images or estimate depth values by obtaining disparity with two or more camera lenses. For example, the initial depth estimation relied on handcrafted features to estimate depth through stereo matching and calibration, which had a disadvantage in that the performance changed significantly depending on the difficulty of image processing, such as illumination and color temperature.

With the recent development of hardware, depth estimation, a deep learning method, is developing with high computing power. However, while wide-field and general-purpose depth estimation is possible using high computing power and a transformer-based encoder, it is still difficult to draw a detailed depth map by highlighting local characteristics. The majority of transformer-based encoders utilize a self-attention mechanism, where self-attention performs attention operations between a reference point, typically a pixel in an image, and all other pixels within the image. Consequently, due to attention operations spanning all regions of the image, there is a risk that local details may not be accentuated, and nuanced depth representations may be obscured by global features.

Therefore, in this paper, an hourglass neck is placed between the encoder and decoder to strengthen the local feature map. Deformable attention is applied in the middle of the neck module to focus more on local areas and extract more features. Since local features are emphasized in the neck module, which extracts and proposes a global feature map from the transformer stage of the encoder, this can help estimate the depth map, which is more distinct and has a clear perspective, such as distinguishing between objects with differences in depth and background. When monocular depth estimation was performed using the hourglass neck module proposed in this paper, the RMSE was calculated to be 0.274. It can be seen that the result is improved compared to the RMSE value of 0.287 without using the hourglass neck module. This paper presents the related works in Section 2, a description of the proposed method in Section 3, the results derived from the proposed method in Section 4, and a discussion in Section 5.

## 2. Related Works

Recently, the use of a depth estimation method that combines stereo images and deep learning has been increasing, breaking away from the classical depth estimation method. The paper [1], published in 2017, carried out unsupervised stereo matching. This paper applied random initialization to set the initial predicted left disparity and predicted right disparity and performed a consistency check through learning to derive a confidence map. The paper [2], published in 2018, produced a disparity map (depth map) by seamlessly reflecting the overall contextual information of the image through CNN [3,4,5] and spatial pyramid pooling (SPP) as an approach to stereo matching, but the edges of objects had a lack of detailed representation.

On the other hand, the monocular depth estimation (MDE) field, which estimates depth values from images acquired from monocular lenses, began to be adopted starting with a paper [6] published in 2014. Depth was estimated from a single image using two network stacks that subdivide their results locally. However, unlike stereo images, MDE has difficulties with estimating the depth value only with local short information; therefore, research is underway to build the depth and structure of the network used in the learning model.

A paper [7] published in 2017 proposed a method for inferring right RGB images from left RGB images, deviating from the existing perspective. A depth map was estimated by calculating the disparity between the inferred right RGB image and the input left RGB image. Similarly, a paper [8] published in 2019 proposed a method for obtaining the disparity between one frame and the next frame by conducting learning using continuous frames in an image sequence without using a ground-truth dataset.

On the other hand, a method for improving the quality of the estimated depth map by combining unsupervised learning Cycle-GAN and segmentation was proposed in a paper [9] published in 2020. This paper suggested that MDE is also possible with unsupervised learning.

Over the past few years, much progress has been made in the field of MDE in reducing the error rate of the estimated depth map and at the same time estimating or generating a depth map similar to the actual ground truth. It could be argued that the paper [10] proposed by Jin Han Lee et al. in 2019 presents an example of using CNN-based supervised learning for MDE to derive compliance results. DeepLab’s atrous spatial pyramid pooling (ASPP) [11], which has the structure of an encoder–decoder and can respond well to multi-scale deep networks to widen the receptive field and enhance the features of the detailed parts of the image, was applied. For the decoder, a local planar guidance (LPG) layer was proposed, which effectively establishes a direct and explicit relationship between the feature extracted from the encoder and the final output. In 2022, a paper by Zhenyu Li et al. [12] sought to improve both the global and local features extracted from the encoder through the HAHI (hierarchical aggregation heterogeneous integration) module. The HAHI module consists of a self-attention module for the enhancement of features obtained from hierarchical layers of the Swin Transformer and a cross-attention module for affinity modeling of features obtained from two heterogeneous encoder branches. The learning model in the paper using the HAHI module consists of an encoder–HAHI module–decoder structure, and the encoder consists of two branches: a Swin Transformer branch and a CNN branch. The feature maps extracted from these two encoder branches were matched in the HAHI module, and different heterogeneous features interacted and cross-attention was performed, producing good results. Meanwhile, in 2023, a paper [13] published by Zhenda Xie et al. introduced masked image modeling (MIM) into depth estimation. MIM is a theory proposed to improve performance in various general-purpose tasks by using the masked image as the transformer’s pre-training data in a situation where the transformer is rapidly emerging as an encoder in various fields. The authors applied the pre-trained model published in SimMIM [14] to the depth estimation task to derive the results of compliance.

In this section, each of the previously introduced methods has been progressively improving accuracy and generating high-quality depth maps. However, it is discerned that there are areas where enhancements can be made. The potential improvements for each system can be summarized as shown in Table 1.

The reasons for suggesting the hourglass neck module in this paper are as follows. First, in a transformer-based encoder with a self-attention module, the globality of the feature map is emphasized and learned, while the locality of the feature map may be relatively insufficient. However, by improving the locality of the feature map using the hourglass neck module, the overall estimation accuracy of the depth map can be improved. Second, the hourglass neck module applies deformable attention to the middle part. In addition, it is a simple form that applies two convolution layers each before and after deformable attention. This structure can refine the feature map extracted from the encoder with lower computational cost and higher locality than self-attention.

## 3. Depth Estimation Method Using Hourglass Neck

**Figure 1:**
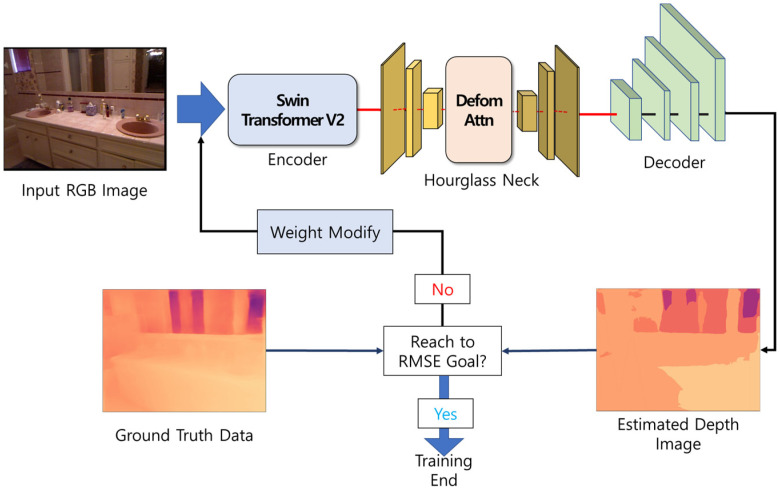
Outline of the proposed depth estimation method; The RGB image passes through an encoder composed of the Swin Transformer V2 to extract a feature map. The extracted feature map is enhanced with local features through the hourglass neck module. The feature map with reinforced local features is input to the decoder to estimate the depth map. The calculated RMSE between the estimated depth map and the ground truth is computed. The training continues by adjusting the weights until the calculated RMSE reaches the target value.

**Figure 2:**
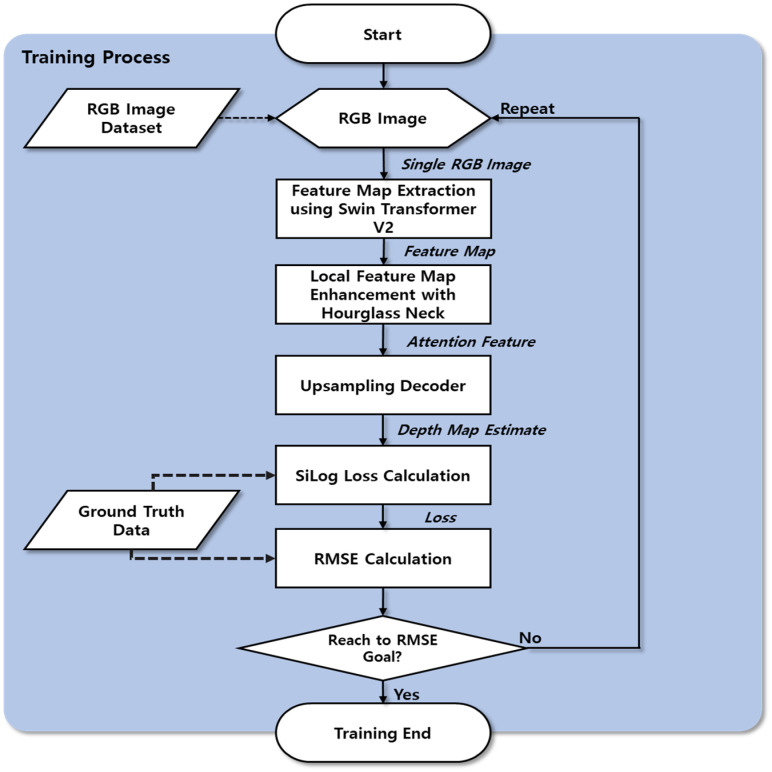
Training process for depth estimation using the hourglass neck module.

Figure 1 outlines the proposed depth estimation method using the hourglass neck module. The training process is shown in Figure 2.

First, after loading SimMIM’s pre-trained masked image modeling learning model, a feature map for the input RGB image is extracted using Swin Transformer V2 [15]. The attention value maintains a stable value because it performs an operation that is not dependent on the amplitude of the input transformer block using scaled cosine attention.

Second, the feature map extracted from the encoder is delivered to the hourglass neck to strengthen the local feature map. The middle of the neck module consists of deformable attention, which allows it to focus on local areas and extract more features. After extracting the global feature map from the encoder’s transformer stage, the neck module emphasizes local features, which can help estimate the depth map with clearer boundaries and more perspective, such as distinguishing between objects with differences in depth and background.

Third, the depth map is estimated by inputting the extracted feature map into the decoder. Since the estimated depth map needs to be compared with the ground truth, the estimated depth map increases to 480 × 480 pixels, which is the spatial size of the input data to the encoder of the learning model. Fourth, a comparison is performed with the estimated depth map and the ground truth of the training dataset to calculate the scale-invariant Log (SiLog) Loss that reduces loss when the distance between the two pixels on the estimated depth map is similar. 

Finally, the estimated depth map is evaluated by calculating the root mean square error (RMSE) between the estimated depth map and ground truth. Learning proceeds in the direction of making both the calculated SiLog Loss and RMSE close to zero. 

Section 3 consists of four subsections, Section 3.1 explains feature map extraction using swin transformer v2, Section 3.2 explains local feature map enhancement with hourglass neck, Section 3.3 explains upsampling decoder, and Section 3.4 explains SiLog loss and RMSE calculation.

### 3.1. Feature Map Extraction Using Swin Transformer V2

**Figure 3:**
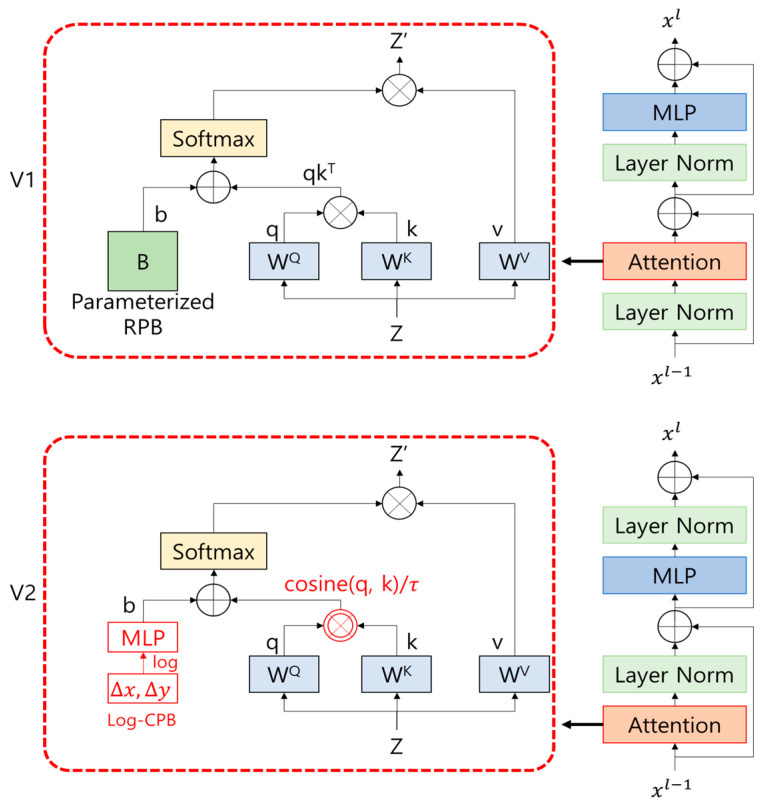
The difference in the block between Swin Transformer V1 and V2; The position of the normalization layer has been moved behind the attention layer. The self-attention operation has been changed to scaled cosine attention. Instead of relative coordinates B, Log-CPB and MLP have been added.

The Swin Transformer is a deep learning network created for image learning, and it performs well, reducing the computational cost of performing self-attention by all patches, which are disadvantages of the existing ViT. ViT makes each patch size 16 × 16 to maintain a total of (224/16)2 = 196 patches, while the Swin Transformer takes the approach of merging more and more patches from a small 4 × 4 patch size like a pyramid structure. Swin Transformer V2 is an improved network to improve the Swin Transformer to be applied to very large images and to effectively utilize pre-trained models learned with small-sized models for transfer learning. Figure 3 shows the difference in the block between Swin Transformer V1 and V2. 

In Figure 3, Z, q, k, and v represent the input feature, query, key, and value in order, and $W^{Q}$, $W^{K}$, and $W^{V}$ refer to the attention weight of the query, key, and value. In addition, ${qk}^{T}$ in V1 is the attention result of the query and key, and this attention operation consists of a matrix multiplication operation. Furthermore, instead of adding absolute coordinates in the existing position embedding, the relative coordinate B is entered into Softmax. In V2, scaled cosine attention is applied instead of the existing attention to perform cosine operations on query and key. After that, it is scaled with a trainable scalar τ. The authors of Swin Transformer V2 posit right after Equation (2), Section 3.2 in their paper, that the scalar τ is not shared between the attention head and layers, and it is set to 0.01 or higher. Instead of relative coordinates B in V1, Log-CPB refers to the relative bias that allows learning to take place well in various window resolutions. The corresponding value is entered into the MLP, the final bias is output, and it is entered into Softmax with the scaled cosine attention result.

**Figure 4:**
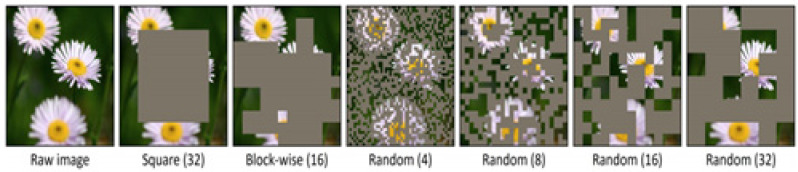
Masking strategy of masked image modeling. The numbers of each method represent the patch size.

In addition, by moving the normalization layer from the beginning to the end of each residual unit, the activation value is lowered, and by using scaled cosine attention instead of the existing self-attention, the operation is performed regardless of the input amplitude, so the attention value remains stable. In this paper, the pre-trained model of MIM is loaded and applied to the model to learn the weight of the image. As shown in Figure 4, when pretraining is performed on an image with a mask between objects and a mask between the object and the background, the original signal (part of image) of the masked area is predicted. This can have the effect of increasing the boundary line prediction bias from the masked area in performing the monocular depth estimation (MDE). Therefore, these allow the MDE to be accurately performed in more diverse environments. Due to the recent computing costs rising continuously, the demand for learning data is increasing, and the depth of the learning model network is deepening. This further highlights the importance and effectiveness of using the pre-trained model, and in this work, a learning model was constructed using the SimMIM pre-trained model.

Masked image modeling (MIM) is the task of masking and predicting a portion of an input image. Random masking is performed on image patches, and the patch size is 32 × 32. The masking ratio is set randomly from 10% to 70%, and a raw pixel regression task is applied to predict a raw image from the masked image.

**Figure 5:**
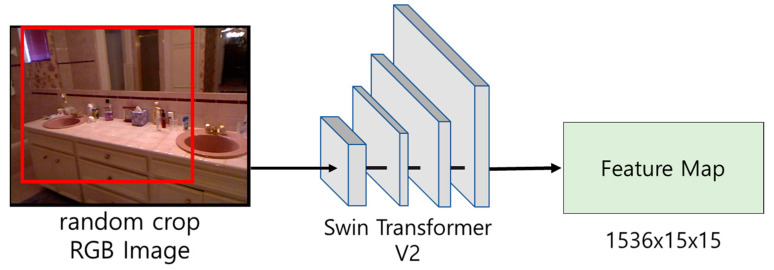
Feature map extraction process using Swin Transformer V2. The red square represents the randomly cropped part of the image.

In this paper, a feature map of size 1536 × 15 × 15 (dimension × height × width) is extracted from RGB images randomly cropped to 480 × 480 using Swin Transformer V2 consisting of four stages. The reason for applying a random crop is not to lose diversity in learning and to reduce the amount of computation. Figure 5 shows the feature map extraction process using Swin Transformer V2. 

Table 2 shows the structure of Swin Transformer V2 used in this paper. Batch size (BS) refers to the number of data samples the learning model trains on at one time. Therefore, assuming that the size of one image is 3 × 480 × 480 and BS is 5, the amount of data input to the learning model at one time is 5 × 3 × 480 × 480.

### 3.2. Local Feature Map Enhancement with Hourglass Neck

**Figure 6:**
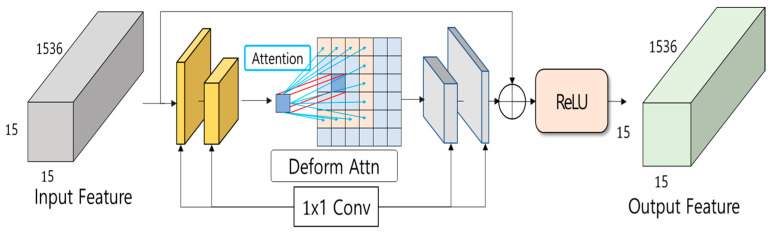
Structure diagram of the hourglass neck module used in this paper.

The hourglass neck module is a neck module in the form of an hourglass and is proposed to strengthen the locality [16] of the feature map extracted from the encoder’s Swin Transformer V2. The transformer has the advantage of being able to use the weight of the pre-trained model using a large amount of datasets and being able to grasp global features well, and it can also be applied to general tasks. However, most transformer blocks are made based on self-attention; the reference point performs attention operations on all pixels in the image. Therefore, there is a disadvantage of not being able to utilize the locality of the feature map well. Therefore, in this paper, the locality of the local feature map is highlighted by proposing an hourglass neck module, with aim of contributing to the accuracy of the final estimated depth map. Figure 6 shows the structure of the hourglass neck module.

**Figure 7:**
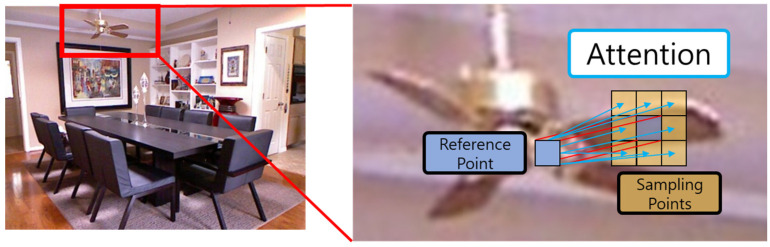
The concept of the hourglass neck module creates a clear difference between the object and the background.

Additionally, Figure 7 shows that the deformable attention of the hourglass neck module can help express clear boundaries and distinguish objects from the background when estimating the depth map. To give a brief example of the concept, assume that there is a reference point expressed as a blue square at the center of the ceiling fan blade to perform deformable attention. Deformable attention performs an attention operation only on sampling points around this reference point. If deformable attention is performed on the edge of an object in this way, the attention score of the object and the surrounding background is bound to be significantly different from self-attention that calculates the entire image.

The hourglass neck module has the same input and output size as BS × 1536 × 15 × 15 (BS × D × H × W) so as not to lose the global features of the feature map extracted from Swin Transformer V2. First, to reduce the computational cost [17], the dimension is reduced to BS × 384 × 15 × 15 using the 1 × 1 convolution layer twice. This can be expressed as Equation (1).

(1) $$x_{conv}=ReLU(Conv\left(\frac{H_{x}}{4},\;\frac{W_{x}}{4},\;Conv\left(\frac{H_{x}}{2},\;\frac{W_{x}}{2},\;x\right)\right))$$

In Equation (1), x is the initial input feature map, and $H_{x}$ and $W_{x}$ are the height and width sizes of the input feature, respectively. Therefore, $Conv\left(\frac{H_{x}}{2},\frac{W_{x}}{2},x\right)$ means that a 1 × 1 convolution operation is taken with a size in which the output height and width are half of the input feature map x.

By applying deformable attention to the center of the neck, the dimension of the input reduced feature map is effectively modeled according to the guidance of the important region. On the other hand, the equation of deformable attention used in this paper is shown in Equation (2).

(2) $$DeformAttn\left(z_{q},p_{q},x\right)=\sum_{m=1}^{M}W_{m}[\sum_{k=1}^{K}A_{mqk}\;\cdot \;W_{m}^{\prime }x(p_{q}+∆p_{mqk})]$$

In Equation (2), x is the input feature map, q is the query element which is a weight vector for the image pixel that is the subject of analysis, $z_{q}$ is the content feature of query element q, $A_{mqk}$ indicates the $k^{th}$ attention weight in the $m^{th}$ attention head, $W_{m}^{\prime }$ and $W_{m}$ are learnable weight $p_{q}$ is the reference point of query element q, m is the attention head index, k is the sampling point index, and $∆p_{mqk}$ is offset to add to the reference point; therefore, $p_{q}+∆p_{mqk}$ becomes a sampling point. $p_{q}+∆p_{mqk}$ is fraction, bilinear interpolation is applied. Both $∆p_{mqk}$ and $A_{mqk}$ are obtained through linear projection of query feature $z_{q}$. $z_{q}$ is supplied to the linear projection operator of 3MK channels. The first 2MK channels encode the sampling offset $∆p_{mqk}$, and the remaining MK channels are fed to the softmax operator to obtain the attention weight $A_{mqk}$. Using Equation (2), the feature map reduced in dimensions in Equation (1) is input into deformable attention. This is expressed as Equation (3).

(3) $$x_{deAttn}=ReLU(DeformAttn\left(z_{q},p_{q},x_{conv}\right))$$

In Equation (3), the deformable attention result derived from Equation (2) passes through the ReLU function and becomes the feature map $x_{deAttn}$. After that, the dimension is expanded twice again using the 1 × 1 convolution layer. The feature map extracted by performing deformable attention is used as an input, which is expressed as Equations (4) and (5). In Equation (4), $2\times H_{x_{deAttn}}$ and $2\times W_{x_{deAttn}}$ are the Height and Width, respectively, derived from the input feature map $x_{deAttn}$ passing through the deconvolution layer.

(4) $$x_{Deconv}=ReLU(Deconv\left(2\times H_{x_{deAttn}},2\times W_{x_{deAttn}},x_{deAttn}\right))$$

(5) $$x_{out}=ReLU(Deconv\left(4\times H_{x_{deAttn}},4\times W_{x_{deAttn}},x_{Deconv}\right))$$

Finally, before inputting into the hourglass neck module for the first time, the feature x and the neck module operation result $x_{out}$ are summed and input to the ReLU function. This is as shown in Equation (6).

(6) $$out=ReLU(x_{out}+x)$$

In this work, in order not to lose the globality of the feature map extracted from Swin Transformer V2, the first tensor input to the hourglass neck is cloned and the sum operation is performed with the output tensor after the operation of the hourglass neck is completed. Finally, after entering the result of the sum operation into the ReLU function, the result value is transferred to the decoder. The ReLU function is a nonlinear activation function that outputs positive values as they are and negative values as zero, with homogeneity but no additionality. By outputting a negative value as 0, the operation is executed faster, and the convergence speed of Loss is very fast because the output value range is wide. 

### 3.3. Upsampling Decoder

**Figure 8:**
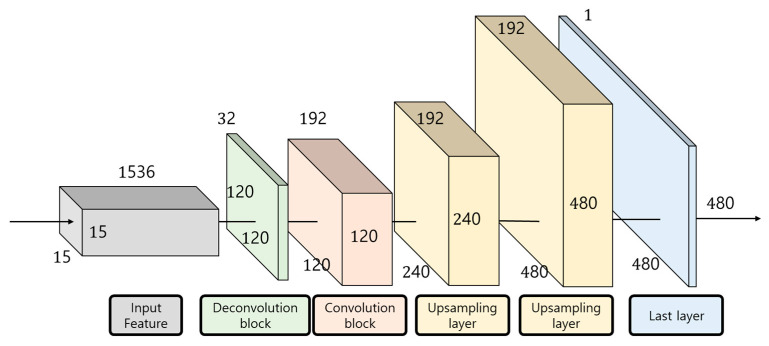
Structure of the decoder used in this paper.

The decoder is composed of a universal deconvolution layer, convolution layer, and upsampling layer. Figure 8 shows the structure of the decoder used in this paper.

The decoder used in this paper is based on the structure of the paper [18] proposed by Kim Doyeon et al. First, the input feature map of BS × 1536 × 15 × 15, which has passed through the Hourglass Neck module, is input into the Deconvolution block. The deconvolution block consists of three layers, and when passing through the block, the map is reduced in dimension to BS × 32 × 120 × 120 × 120, and the size increases. Then, using the convolution layer, the height and width of the feature map are fixed, and the dimension is extended to 192 only. This is because if expansion to the same size as the image input rapidly to the initial model is attempted while the dimension is expended, there is a possibility that the feature map with the reduce the density of meaningful data [19]. The feature map, which passes through the convolution layer, sets the scale factor to 2 and passes through the upsampling layer twice to restore the size of HxW to the same size as the initial model. The final depth map estimation is then performed through the last Conv-ReLU-Conv-Sigmoid layer.

### 3.4. SiLog Loss and RMSE Calculation

In this paper, the scale-invariant Log (SiLog) Loss function is used as the Loss function. The SiLog Loss function compares the estimated depth map with the ground truth of the training dataset to reduce loss when the distance between the two pixels on the estimated depth map is similar to the ground truth. Equation (7) represents the scale-invariant Log Loss function.

(7) $$L\left(y,y^{*}\right)=\frac{1}{n}\sum_{i}{\left\{\log\left(y_{i}\right)-\log\left(y_{i}^{*}\right)\right\}}^{2}-\frac{\lambda }{n^{2}}{\left\{\sum_{i}\log\left(y_{i}\right)-\log\left(y_{i}^{*}\right)\right\}}^{2}$$

In Equation (7), n is the total number of pixels, and i is the index of the corresponding pixel. $d_{i}$ is the log($y_{i}$) applied to the estimated depth map minus the log($y_{i}^{*}$) applied to the depth map, which is the ground truth. Referring to Equation (4) of the paper proposed by Eigen et al. [6], λ is set to 0.5 to operate as a loss function with scale invariance.

Meanwhile, the root mean square error (RMSE) is used to evaluate the estimated depth map. The equation of RMSE is shown in Equation (8).

(8) $$RMSE=\sqrt{MSE}$$

The RMSE function is a function that puts the root on the mean square error (MSE) function. The equation of the MSE function is shown in Equation (9).

(9) $$MSE=\frac{1}{n}\sum_{i=1}^{n}{\left(y_{i}-y_{i}^{*}\right)}^{2}$$

In Equation (9), $y_{i}$ is the predicted value of the pixel *i* estimated depth map, and $y_{i}^{*}$ is the real value of pixel *i* ground-truth. *n* is the total number of pixels of the depth map. In addition, since the dataset usually dealt with in deep learning is vast and often large, the value of MSE sometimes becomes too large when the error agreement value is calculated to be very large. Therefore, for reasons such as a decrease in computational speed, the RMSE function that puts the root on the MSE function is used instead of the general MSE function. Learning is continued by updating the weight values until the RMSE and SiLog functions reach the target value.

**Figure 9:**
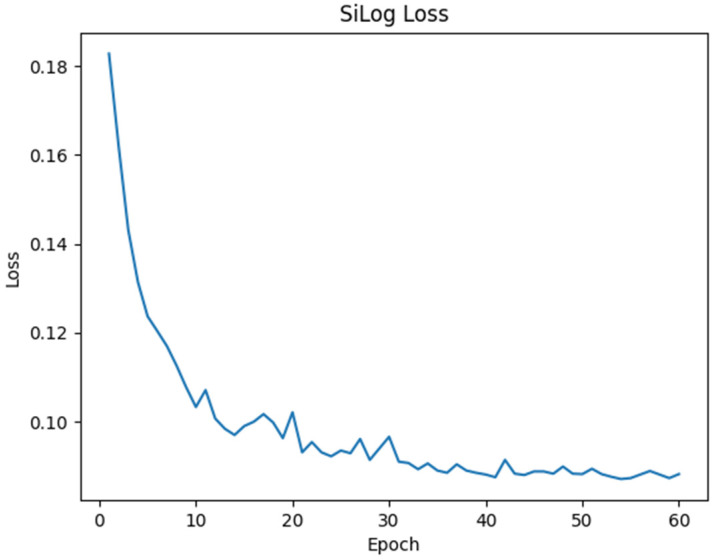
Graph showing the change in SiLog Loss values for the NYU Depth V2 dataset.

Figure 9 is a loss graph of the SiLog function calculated while learning Epoch 60 using the NYU Depth V2 dataset [20]. The system learned 24,231 images pair, while 654 pair test sets were used for validation.

The code corresponding to this paper can be found at the following address and access on 2 February 2024: https://github.com/TGFLOPS/Hourglass-MDE.

## 4. Results

To evaluate the objective reliability of the proposed method in this paper, the experiment was conducted using the NYU Depth V2 dataset. The RGB image is retrieved from the NYU Depth V2 dataset and entered into an encoder consisting of Swin Transformer V2 to extract the feature map. The extracted feature map reinforces locality using the hourglass neck module. After that, the SiLog Loss and RMSE for the estimated depth map are calculated by entering the upsampling decoder to calculate the result.

**Figure 10:**
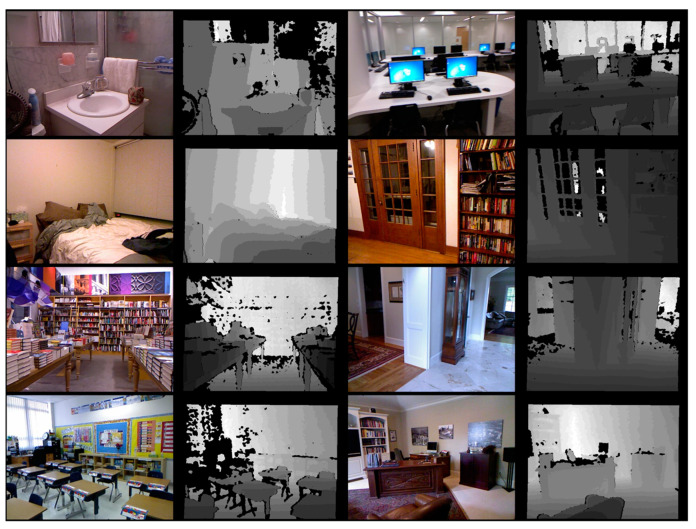
Example test set of NYU Depth V2 dataset used in this paper.

**Figure 11:**
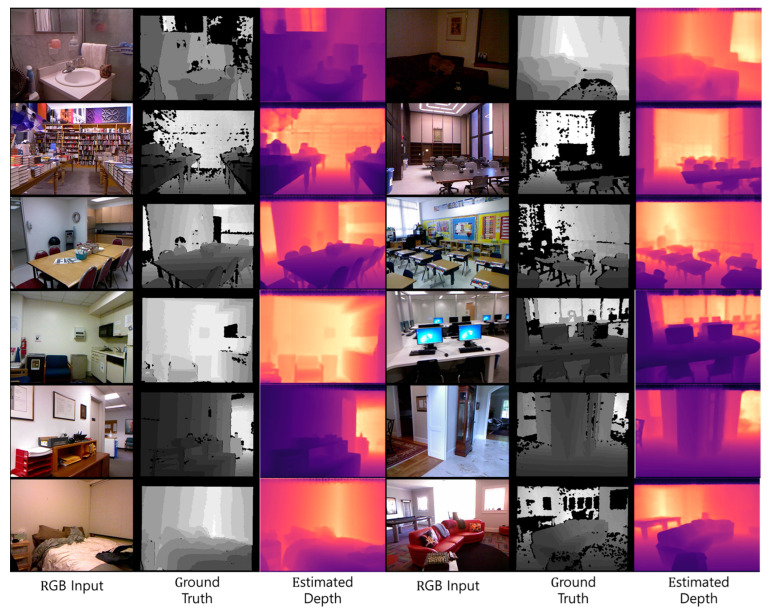
Depth estimation results of the images from the NYU Depth V2 dataset.

The NYU Depth V2 dataset was a standard dataset built and released by New York University with Kinect camera from Microsoft, Washington, USA, which was used as a comparative benchmark in existing papers. The NYU Depth V2 dataset consists of a training set of 24,231 RGB and depth ground-truth images pair in 26 indoor locations and a test set of 654 RGB and depth ground-truth images pair. In this paper, 654 test sets of 16 categories were used as shown in Table 3 to construct and evaluate the same environment based on the results performed in comparative papers. Figure 10 shows an example image of the NYU Depth V2 dataset used in this paper, and Figure 11 shows the depth map estimated using the proposed method and the NYU Depth V2 dataset. In Figure 11, the black area at the edge of the ground truth photo taken with a kinetic camera is caused by the disparity in the ground truth data between the left and right lenses of the camera. The estimated depth figures are the result of normalizing and colorizing the original depth map generated by the learning model for visibility.

The hardware used in the experiment described in this paper consists of Intel(R) Xeon(R) Silver 4214R 2.4 GHz CPU, 128 GB of RAM, and NVIDIA GeForce RTX A6000 (VRAM 48 GB) GPU. Experiments were conducted using the Ubuntu 20.04 operating system, using Visual Studio Code and Python 3.8.10. The main libraries used were CUDA 11.3, cuDNN v8.4.1, Pytorch 1.11.0, etc.

In order to evaluate the objective performance of the monocentric depth estimation using the hourglass neck module proposed in this paper, we compared and evaluated the methods published in other papers using the NYU Depth V2 dataset. RMSE was used as an accuracy evaluation metric. Table 4 shows the comparison results of the NYU Depth V2 dataset between the proposed method in this paper and the methods published in other papers, and the RMSE was adjusted to three decimal places. The proposed method performed monocular depth estimation by applying the hourglass neck module and produced excellent results with an RMSE of 0.274. The absolute relative error (AbsRel) also means that the lower the value, the better the quality of the estimated depth map. The δ1, δ2 and δ3 values indicate a better depth estimation method: the higher the better. δ1, δ2 and δ3 metrics represent the ratio between the larger and smaller values among the predicted and true values. In this context, a threshold is employed, and if the ratio is smaller than the threshold, it is considered a True Positive. In the majority of MDE papers, the threshold values are denoted as δ1 = 1.25, δ2 = $1.25^{2}$, and δ3 = $1.25^{3}$. Table 5 shows the results of not applying monocular depth estimation and the hourglass neck module with the hourglass neck module. Furthermore, we measured the total number of parameters in the learning model when utilizing and not utilizing the Hourglass Neck module to compare computation costs. The utilization of the Hourglass Neck module yielded improved results, and the increase in the number of parameters was marginal.

The arrow symbols in Table 4, Table 5 and Table 6 indicate the directionality of each evaluation metric. If the arrow points downward, it signifies that a lower value of the corresponding metric reflects better performance of the learning model. Conversely, if the arrow points upward, it indicates that a higher value of the evaluation metric represents superior performance of the learning model.

**Figure 12:**
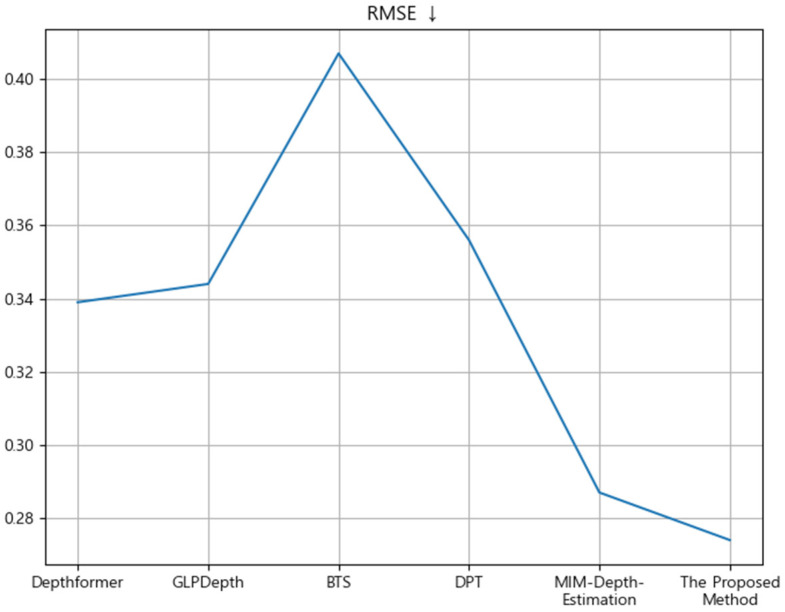
Comparison of the RMSE value of the proposed method and the RMSE values of methods presented in other papers on images from the NYU Depth V2 dataset.

Figure 12 presents a graph showing the results of the comparative evaluation of the RMSE. The RMSE value of the proposed method was lower than that of the methods published in other papers. The RMSE value indicates that the closer the value is to zero, the better it is compared to the methods published in other papers. 

**Figure 13:**
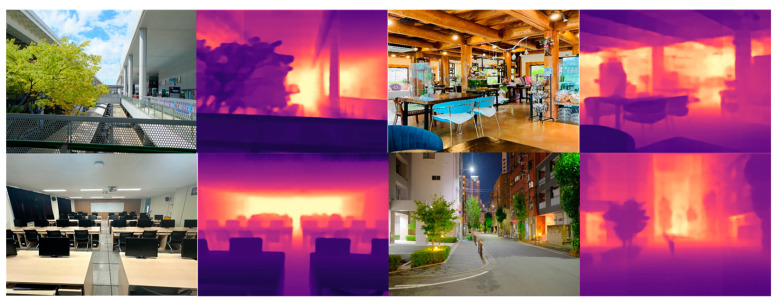
Estimated depth map from real-life pictures using the proposed method.

**Figure 14:**
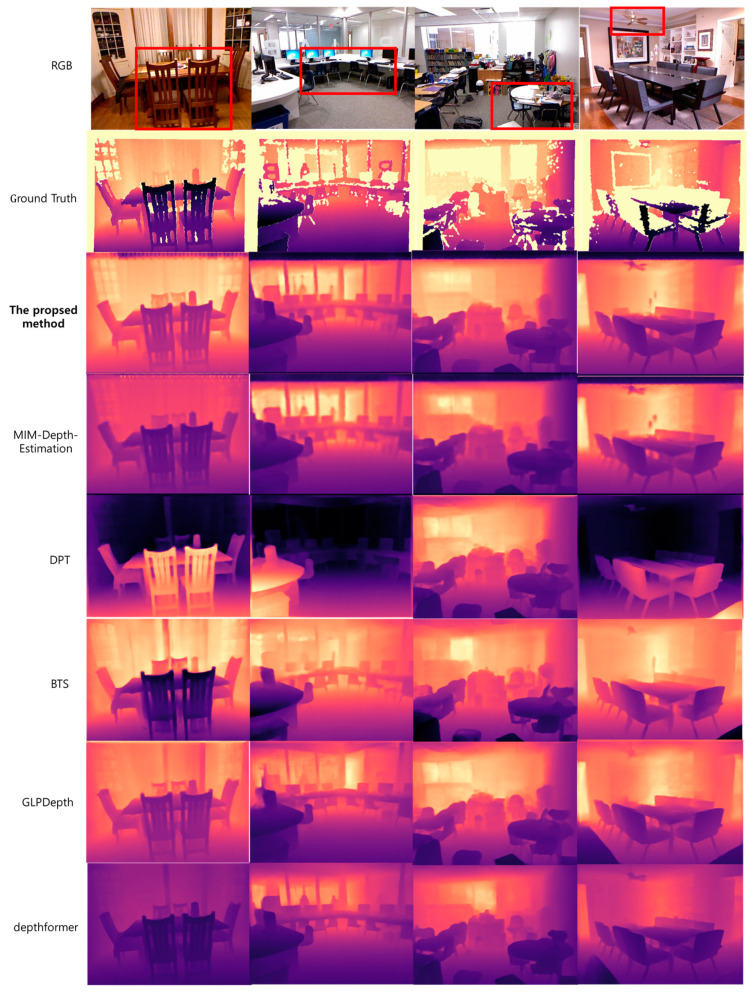
Comparison results of the estimated depth map with the proposed method and methods in comparative papers. The red square in the RGB image constitutes an area wherein the juxtaposition of intricate objects facilitates a nuanced comparison of localized feature points within indoor imagery.

Figure 13 presents the result of estimating the depth map from real-life photos rather than the NYU Depth V2 dataset using the proposed method; Figure 14 shows the result of comparing the method in Table 3 with the local depth estimation. It can be seen that the local depth estimation of the proposed method, as indicated by the red box, performed well.

Additionally, to substantiate the improvements of the proposed method, we conducted comparisons by focusing solely on the region corresponding to the red box in Figure 14, comparing it with the ground truth. The results for this analysis are presented in Table 6. Due to the reduction in size of the evaluation region compared to the original NYU Depth V2 dataset images, there may be variations in the scale of each metric result value. Also, the corresponding input data in Table 6 is the result of comparing the png files of the Depth Map output from each method. However, both methods were compared against the same region of the ground truth.

The following and final section, Section 5, briefly explains the methodology of this paper and future research directions.

## 5. Discussion

In this paper, a novel method for monocular depth estimation using the hourglass neck module was proposed. The proposed method extracts a feature map from Swin Transformer V2 using the MIM pre-trained model. Swin Transformer V2 has a different patch size for each attention stage, so it is easier to extract local and global features from images input by the vision transformer (ViT)-based encoder. To refine and maintain the polymorphism and local inductive bias of the feature map extracted from the attention head of Swin Transformer V2, feature maps are passed through the hourglass neck to aid model learning. In addition, deformable attention can be used in the middle of the hourglass neck module to reduce the computational cost and highlight the locality of the feature map. The feature map passing through the neck passes through a decoder consisting of a deconvolution layer and an upsampling layer to estimate the depth map. The accuracy of the method proposed in this paper and those published in other papers was compared. In order to evaluate the objective reliability of the comparison results, the NYU Depth V2 dataset, which is a published standard dataset, was used for experimentation. The RMSE value of the method proposed in this paper was calculated as 0.274, and the lower the value, the better the result, so the superior efficiency of the performance was proven in the results of monocular depth estimation compared to that of the methods published in other papers.

On the other hand, the proposed method exhibited improved results compared to existing methods, albeit marginally, and incurred a slight increase in computation cost. Minimizing the increase in computation cost while finding the threshold that maximally enhances the performance of the learning model seems crucial.

According to recent publications, achieving highly satisfactory results involves additional training on a significantly large scale of unlabeled data [22] or incorporating semantic segmentation [23]. The outcomes are very promising; however, as the models and data in these papers are progressively increasing in size, the computation cost is also on the rise. Without additional data, it seems plausible to anticipate improvements in results by applying semantic segmentation to the existing Swin Transformer V2-based learning model network used in this paper. As for the future research direction, it is considered necessary to estimate the depth map without relying too much on the weight on the feature map produced by the transformer. The reason is that only Swin Transformer V2 was used for the encoder in this paper, and the transformer requires a large quantity of learning data to learn to perform above the threshold. Therefore, the pre-trained model is essential because the weight of the MIM pre-trained model is too large to detect the local feature map well. In addition, research is needed to increase the accuracy of the monocular depth estimation results to the actual measurement level. Finally, research in the relevant field is needed so that it can be combined with fields such as AR and VR through the depth map estimated in this paper.

## Figures and Tables

**Table 1:** Possible improvements for each introduced method.

Method	Possible Improvements Points
Unsupervised learning of stereo matching [1]	Dependency on initial predicted disparity
Pyramid stereo matching network [2]	Lack of detailed object representation
Digging into self-supervised monocular depth estimation [8]	Operability of large and heavy networks
Cycle-GAN with segmentation [9]	Relatively long learning time, a characteristic of unsupervised learning
BTS [10]	Dependency on decoder
Depthformer [12]	Complexity of calculations
Depth estimation with masked image modeling [13]	Lack of detailed object representation

**Table 2:** Structure table of Swin Transformer V2 applied in this paper.

Stage	Contents	Count	Output Size
0	Input RGB Image	X Batch Size	BS, 3, 480, 480
1	Linear Embedding	X1	BS, 14,400, 192
	Swin Transformer Block	X2	
2	Patch Merging	X1	BS, 3600, 384
	Swin Transformer Block	X2	
3	Patch Merging	X1	BS, 900, 768
	Swin Transformer Block	X6	
4	Patch Merging	X1	BS, 225, 1536
	Swin Transformer Block	X2	
-	Normalize	X1	BS, 1536, 15, 15
Swin Transformer Block	Window Multi-Head Attention	X1	-
	Layer Normalization		
	Multi-Layer Perceptron		
	Layer Normalization		
	Shifted Window Multi-Head Attention		
	Layer Normalization		
	Multi-Layer Perceptron		
	Layer Normalization		

**Table 3:** Test set in 16 categories of NYU Depth V2 dataset.

Place	Amount of Pair Data
Bathroom	58
Bedroom	191
Bookstore	11
Classroom	23
Computer lab	3
Dining room	55
Foyer	2
Home office	24
Kitchen	106
Living room	107
Office	38
Office kitchen	4
Playroom	14
Reception room	5
Study	11
Study room	2

**Table 4:** Results of the RMSE for the proposed method and those from other papers on images from the NYU Depth V2 dataset.

Method	RMSE↓	AbsRel↓	δ1↑	δ2↑	δ3↑	#Params↓
Depthformer [12]	0.339	0.096	0.921	0.989	0.998	273 M
GLPDepth [17]	0.344	0.098	0.915	0.988	0.997	62 M
BTS [9]	0.407	0.110	0.885	0.978	0.994	47 M
DPT [21]	0.356	0.110	0.904	0.988	0.998	225 M
MIM-Depth-Estimation [13]	0.287	0.083	0.949	0.994	0.999	148 M
The Proposed Method	0.274	0.097	0.953	0.994	0.999	151 M

**Table 5:** Results of the proposed method with and without the hourglass neck module applied.

Method	RMSE↓	AbsRel↓	δ1↑	δ2↑	δ3↑	#Params↓
Without Hourglass Neck Module	0.288	0.087	0.953	0.993	0.998	148 M
The Proposed Method	0.274	0.097	0.953	0.994	0.999	15 1M

**Table 6:** Results of the proposed method for the local region with and without the hourglass neck module applied.

Method	RMSE↓	AbsRel↓	δ1↑	δ2↑	δ3↑
Without Hourglass Neck Module	1.031	0.110	0.934	0.999	0.999
The Proposed Method	1.128	0.125	0.891	0.999	0.999

## Data Availability

The dataset used in this paper can be downloaded from the link follow. (https://cs.nyu.edu/~silberman/datasets/nyu_depth_v2.html).
